# Mitochondrial Respiratory Chain Dysfunction—A Hallmark Pathology of Idiopathic Parkinson’s Disease?

**DOI:** 10.3389/fcell.2022.874596

**Published:** 2022-04-01

**Authors:** Irene H. Flønes, Charalampos Tzoulis

**Affiliations:** ^1^ Neuro-SysMed, Department of Neurology, Haukeland University Hospital, Bergen, Norway; ^2^ K.G Jebsen Center for Translational Research in Parkinson’s Disease, University of Bergen, Bergen, Norway; ^3^ Department of Clinical Medicine, University of Bergen, Bergen, Norway

**Keywords:** neurodegeneration, mitochondrial complex I, mitochondria, oxidative phosphorylation, Parkinson's disease

## Abstract

Parkinson’s disease (PD) is the most common age-dependent neurodegenerative synucleinopathy. Loss of dopaminergic neurons of the substantia nigra pars compacta, together with region- and cell-specific aggregations of α -synuclein are considered main pathological hallmarks of PD, but its etiopathogenesis remains largely unknown. Mitochondrial dysfunction, in particular quantitative and/or functional deficiencies of the mitochondrial respiratory chain (MRC), has been associated with the disease. However, after decades of research in this field, the pervasiveness and anatomical extent of MRC dysfunction in PD remain largely unknown. Moreover, it is not known whether the observed MRC defects are pathogenic, compensatory responses, or secondary epiphenomena. In this perspective, we give an overview of current evidence for MRC dysfunction in PD, highlight pertinent knowledge gaps, and propose potential strategies for future research.

## Introduction

Parkinson’s disease (PD) is the most common neurodegenerative synucleinopathy, and affects approximately 1.8% of the population above the age of 65 years (de Rijk et al., 2000). Idiopathic PD is the predominant form of the disease, whereas monogenic forms of PD are rare, generally accounting for <5% of the cases in most populations (Bloem et al., 2021). This work will focus on idiopathic PD, which will be henceforth referred to simply as PD, unless otherwise stated. Region-specific formation of Lewy pathology (LP), of which misfolded and aggregated α -synuclein is a main constituent, and loss of the dopaminergic neurons of the substantia nigra pars compacta (SNc), are the pathological hallmarks of PD (Dickson, 2012; Surmeier et al., 2017). Neuronal loss is not limited to the SNc, but occurs across multiple brain regions, and is commonly accompanied by astro- and microgliosis. In addition to LP, accumulation of tau and beta-amyloid is also commonly observed in PD (Dickson, 2012).

Mitochondria have been the subject of intensive research in PD, following the discovery that chemical inhibition of complex I (CI) of the mitochondrial respiratory chain (MRC) can cause a parkinsonian phenotype in humans and other primates, with dopaminergic neuron loss and inclusions resembling LP in the SNc (Langston et al., 1983; Ramsay et al., 1986; Betarbet et al., 2000). Shortly after, it was demonstrated that CI deficiency occurs in the SNc of individuals with PD. While CI deficiency in the dopaminergic neurons of the SNc has been a consistent finding in subsequent studies of PD, reports from other brain regions are variable and conflicting. A comprehensive review of studies on this topic was recently published by Subrahmanian et al. (Subrahmanian and LaVoie, 2021). Thus, the pervasiveness and anatomical extent of CI deficiency in PD remains a subject of debate, four decades since its first description. Moreover, the etiopathogenesis and downstream impact of CI deficiency in PD are unknown. Here, we provide a brief overview of current evidence, regarding the role of MRC deficiency in PD.

## State of the Art on Mitochondrial Respiratory Chain Dysfunction in Parkinson’s Disease

### Evidence of Mitochondrial Respiratory Chain Dysfunction in Parkinson’s Disease

**FIGURE 1:**
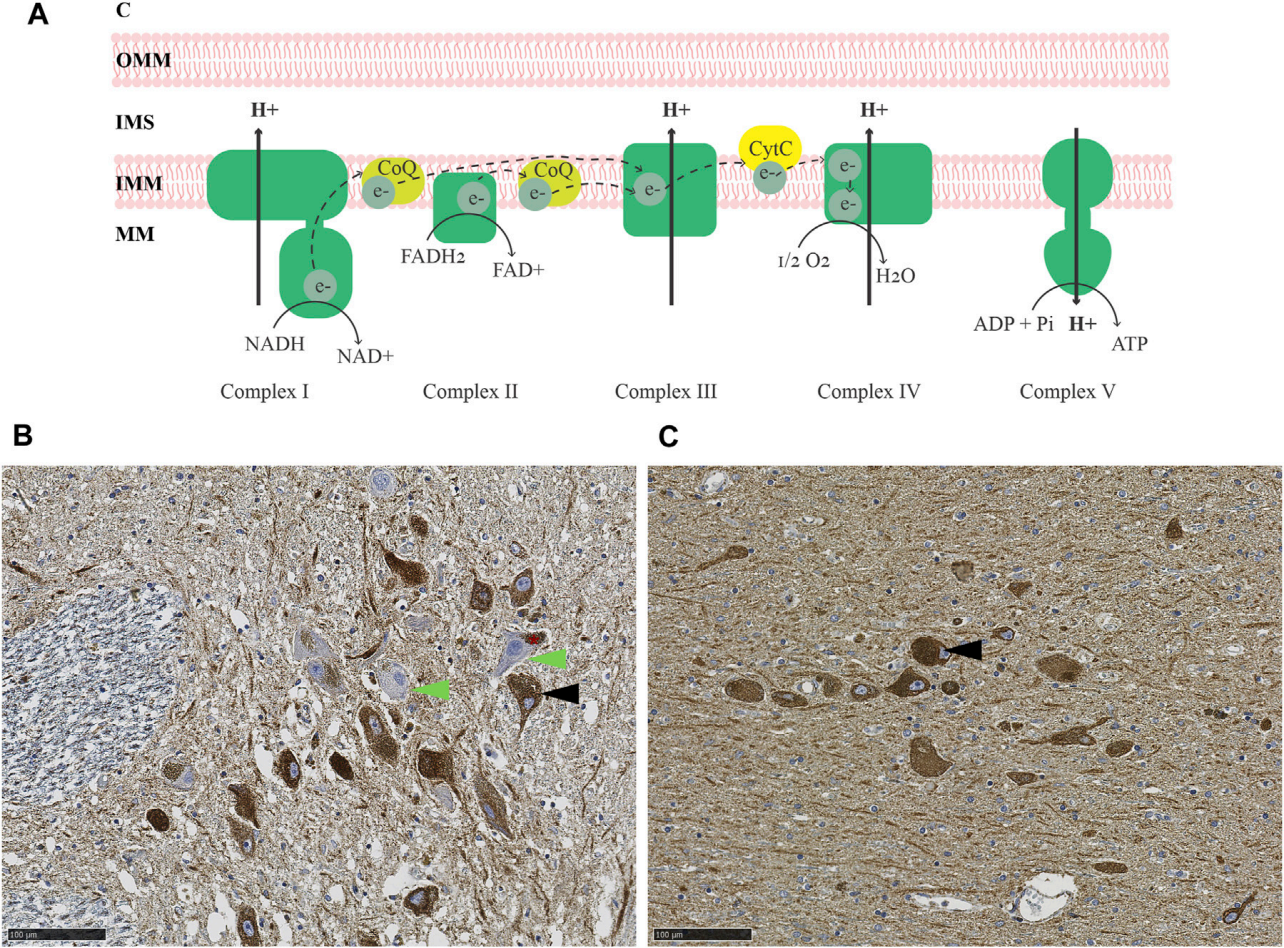
MRC deficiency in PD. **(A)** Schematic representation of the MRC. Electrons are transferred from NADH and FADH_2_ to complex I and II, respectively, and carried by ubiquinol (coenzyme Q; CoQ) to complex III. From there, electrons are further transferred to cytochrome c and transported to complex IV, which finally transfers the electrons onto molecular oxygen, generating water. The energy released during the series of electron transfers and redox reactions is used to pump protons out of the mitochondrial matrix (MM) via complexes I, III, and IV, creating an electrochemical and pH gradient. Protons flow back into the mitochondrial matrix via complex V and this flux is coupled with the phosphorylation of ADP to generate ATP. **(B–C)** Immunohistochemistry for the CI subunit NDUFS4 **(B)** and mitochondrial mass marker VDAC1 **(C)** in the SNc of an individual with idiopathic PD. The asterisk in **(B)** indicates neuromelanin in dopaminergic neurons of the SNc. Arrowheads show examples of CI deficient (green) and intact (black) neurons. Magnification 20X. Scalebar: 100 µm. C: cytosol, OMM: outer mitochondrial membrane, IMS: intermembrane space, IMM: inner mitochondrial membrane, MM: mitochondrial matrix, CoQ: coenzyme Q, CytC: cytochrome C, NADH: reduced nicotinamide adenine dinucleotide, NAD^+^: oxidized nicotinamide adenine dinucleotide, FADH_2_: reduced flavin adenine dinucleotide, FAD^+^: oxidized flavin adenine dinucleotide, O_2_: molecular oxygen, H_2_O: water, ADP: adenosine diphosphate, Pi: phosphate, ATP: adenosine triphosphate.

The brain constitutes approximately 2% of the total body weight, yet it accounts for 20% of the resting metabolic rate (Mary et al., 2012). The majority of ATP synthesis occurs at the MRC, via the process of oxidative phosphorylation (Berg et al., 2007) (Figure 1A). Studies of the MRC in PD have generally relied on two types of methodological approaches, quantitative and functional. Quantitative assays are based primarily on immunodetection, either in bulk, homogenized tissue (e.g., by immunoblot), or *in situ* by immunohistochemistry (IHC) (Subrahmanian and LaVoie, 2021). Functional assays measure the specific activity of each respiratory complex by quantifying substrate conversion, usually in mitochondria isolated from homogenized bulk tissue (Birch-Machin and Turnbull, 2001).

Evidence for both quantitative and functional MRC deficiency has been reported in PD, but findings vary depending on the study and brain region being examined (Subrahmanian and LaVoie, 2021). In the SNc, most studies report evidence of MRC deficiency in PD, affecting mostly CI and, to a lesser degree, complexes II-IV (Subrahmanian and LaVoie, 2021) (Figures 1B,C). That said, the dopaminergic neurons of the SNc exhibit pronounced MRC deficiencies of similar type also with neurologically healthy aging (Bender et al., 2006; Kraytsberg et al., 2006). While subjects with PD generally show more severe deficiency than age-matched controls at the group level, there is substantial overlap at the individual level (Chen et al., 2021), so that disease-specific effects cannot be fully disentangled from the impact of aging.

MRC deficiencies have been reported in regions outside the SNc. However, findings are inconsistent and, in part, contradictory, with several studies showing no significant difference between cases and controls at the group level (Subrahmanian and LaVoie, 2021). Furthermore, the question of whether non-neuronal cells in the central nervous system exhibit MRC dysfunction in PD is only starting to be explored, with one recent study reporting quantitative reduction in all MRC complexes, normalized for total mitochondrial mass, in SNc astrocytes (Chen et al., 2022). Studies in peripheral tissues of individuals with PD, including platelets, skeletal muscle and lymphocytes, have produced similarly conflicting results, with some, but not all, detecting functional and/or quantitative reduction of CI and other MRC complexes, compared to controls (Subrahmanian and LaVoie, 2021).

Thus, while MRC dysfunction, mainly in the form of CI deficiency, is undoubtfully a phenomenon associated with PD, its presence shows high anatomical/regional and individual variation. Methodological differences may partly account for this variability. For instance, studies in homogenized bulk tissue may be less sensitive in detecting cell-specific changes, and prone to bias from altered tissue cell composition. That said, results vary also between studies using cell-specific approaches (Subrahmanian and LaVoie, 2021). This variability raises the pertinent question of whether MRC dysfunction is a universal feature of PD, or rather one characterizing a particular subset of individuals.

### The Origin of Mitochondrial Respiratory Chain Deficiency in Parkinson’s Disease Remains Largely Unknown

A unique feature of the MRC is that it is encoded by two genomes: 13 of its subunits are encoded by the mitochondrial DNA (mtDNA) and the remaining by the nuclear DNA (Mukherjee and Ghosh, 2020). Thus, the question arises of whether variation in any, or both, of these genomes is responsible for MRC dysfunction in PD. Genetic association studies suggest that polygenic variation enrichment in nuclear genes regulating the processes of mtDNA maintenance (Billingsley et al., 2019), mitophagy and mitochondrial translation (van der Walt et al., 2003; Pyle et al., 2005), all of which are critical for MRC integrity and function, is associated with PD. Moreover, inherited variation in the mtDNA itself appears to be important, as certain mtDNA haplogroups have been associated with lower risk of PD (van der Walt et al., 2003; Pyle et al., 2005). The reported associations are, however, relatively weak, and unlikely to be, alone, driving the observed MRC deficit in PD.

Somatic mtDNA changes are an established cause of MRC deficiency and have been associated with PD (Bender et al., 2006). Dopaminergic neurons of the SNc accumulate high levels of somatic mtDNA deletions with age (Bender et al., 2006), and this can reach even higher levels in PD (Dölle et al., 2016). Moreover, while neurologically healthy individuals appear to compensate for the accumulation of age-dependent mtDNA deletions by increasing their total mtDNA copy number, this response is blunted in PD, resulting in loss of wild-type mtDNA (Dölle et al., 2016). In line with this observation, single SNc neurons with MRC deficiencies show both a decrease in mitochondrial transcription factor A (TFAM) expression and total mtDNA copy number in PD (Chen et al., 2020). The role of mtDNA point mutations (i.e., single nucleotide variants, SNV), is more controversial. While some studies report no increase in point mutational load in PD (Simon et al., 2000; Vives-Bauza et al., 2002), others indicate that heteroplasmic point mutations occur in genes encoding subunits of CI (Parker and Parks, 2005) and CIV (Coxhead et al., 2016). Moreover, increased levels of mtDNA point mutations have been reported in single SNc neurons in early PD and incidental Lewy-body disease, indicating that point mutations may be present in early stages of PD (Lin et al., 2012).

An alternative hypothesis is that neuronal mtDNA changes in PD may be the result, rather than the cause, of MRC dysfunction. Under this model, mtDNA deletions and, potentially, point mutations arise during the repair of double-strand breaks, caused by increased reactive oxygen species (ROS) generation in CI deficient neurons (Verkaart et al., 2007; Zsurka et al., 2018). Yet another hypothesis, based on cell culture experiments, proposes that CI loss in PD may be mediated by an intra-mitochondrial LON-ClpP proteolytic quality control axis, which cleaves the peripheral arm of the complex, in response to ROS generation in depolarized mitochondria (Pryde et al., 2016). In this scenario, CI deficiency would be a compensatory response to mitigate ROS-related damage. While an intriguing hypothesis, the role of ROS in the pathogenesis of PD remains highly uncertain (Nissanka and Moraes, 2018).

**FIGURE 2:**
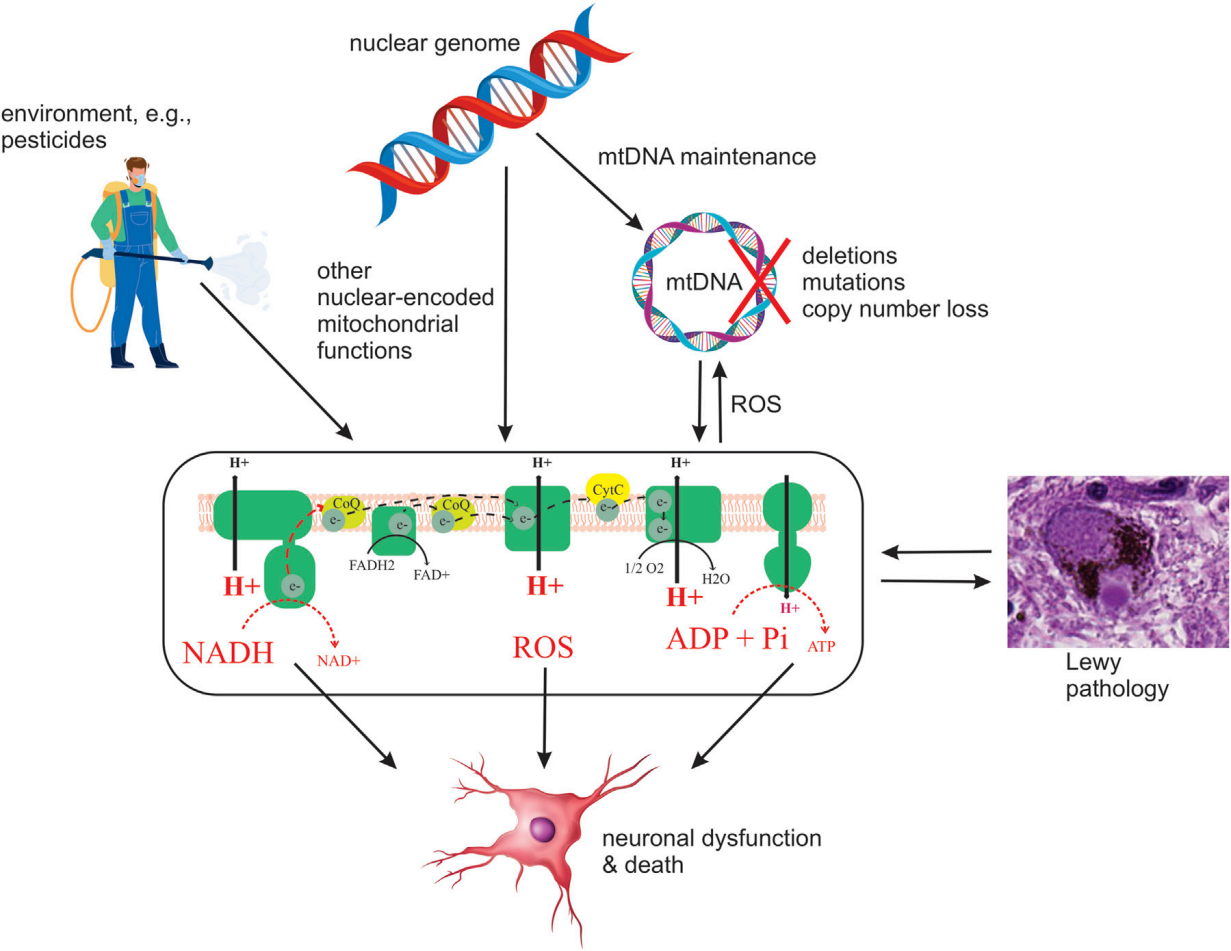
Schematic depiction of current hypotheses on the origin and role of MRC deficiency in PD. Potential upstream etiologies are summarized, including genetic and environmental factors. Four potential downstream consequences of deficient MRC function are shown: decreased oxidation of NADH to NAD^+^ by CI, decreased electron transfer, resulting in decreased proton pumping and, in turn, loss of membrane potential and lower ATP production, and increased generation of ROS. It should be emphasized that this is by no means an exhaustive list of possible causes or consequences of MRC deficiency in PD.

Since heritability only accounts for part of the PD risk (Nalls et al., 2019). It is reasonable to assume that the environment also contributes to the observed MRC dysfunction. The fact that chemical CI inhibition causes SNc degeneration and parkinsonism has led to the hypothesis that chronic exposure to mitochondrial toxins, such as the ones contained in various pesticides, may contribute to MRC dysfunction in individuals with PD (Brown et al., 2006). Indeed, several epidemiological studies have found associations between exposure to pesticides known to inhibit CI and the incidence/prevalence of PD. However, to date, no direct causality has been established (Figure 2). Epidemiologic and toxicologic evidence of association between pesticide exposure and the risk of PD and/or parkinsonism has been reviewed by Brown et al. (Brown et al., 2006).

### Is Mitochondrial Respiratory Chain Dysfunction Linked to α -Synuclein Pathology in Parkinson’s Disease?

The question of whether and how mitochondria and α -synuclein interact in health and disease has been a topic of investigation ever since α -synuclein was recognized as a main constituent of LP, along with mitochondria (Spillantini et al., 1997; Shahmoradian et al., 2019). In its native state, α -synuclein is a monomer of multiple conformational states, which is believed to be involved in the regulation of synaptic vesicle trafficking and neurotransmission (Bobela et al., 2015; Alam et al., 2019). The conformational plasticity of α -synuclein monomers has been suggested to facilitate the formation of fibrils and/or oligomers, which have been suggested to be cytotoxic, although their pathogenic contribution in PD remains uncertain (Alam et al., 2019; Kumar et al., 2020; Mahul-Mellier et al., 2020; Bisi et al., 2021; Outeiro, 2021).

Studies in cell and animal models generally support a link between mitochondria and α -synuclein, but the dynamics of this relationship remains poorly understood. In yeast it was shown that functional mitochondria are required for α -synuclein-induced toxicity and cell death (Büttner et al., 2008). However, several studies in mammalian cells and animals revealed a different picture, suggesting that α -synuclein and MRC dysfunction may act synergistically in PD. Namely, α -synuclein knockout mice were resistant to CI inhibition by parkinsonism-inducing neurotoxins (Klivenyi et al., 2006), whereas mice overexpressing α -synuclein were more vulnerable to the same toxins (Song et al., 2004). Studies in cybrid-derived neurons from mouse fibroblasts carrying pathogenic mtDNA mutations revealed that α -synuclein aggregates inhibit CI function in wild-type and complex IV(CIV)-deficient cells, resulting in increased levels of apoptosis, although they did not exacerbate established CI deficiency (Reeve et al., 2015). Furthermore, it has been shown that aggregated α -synuclein induces mitochondrial dysfunction, including CI deficiency and opening of the permeability transition pore, in co-cultures of rat neurons and astrocytes, as well as in human induced pluripotent stem cells (iPSC)-derived neurons (Ludtmann et al., 2018).

To our knowledge, few studies have explored the link between MRC dysfunction and α -synuclein pathology in the human brain. Two studies revealed an inverse association between neuronal CI deficiency and the presence of LP, in the form of Lewy bodies or pale bodies, in PD and Parkinson’s disease dementia—i.e., neurons deficient for CI were significantly less likely to contain formed LP aggregates (Reeve et al., 2012; Flones et al., 2017). The mechanisms underlying this inverse association are unknown. One possibility is that functional MRC is required for LP formation, similar to what has been shown in yeast (Büttner et al., 2008). Alternatively, the coexistence of MRC deficiency and LP may be additively, or even synergistically, deleterious, so that neurons that suffer a “double hit” are lost early in the disease (Reeve et al., 2012).

### Whether and How Mitochondrial Respiratory Chain Dysfunction Contributes to Neuronal Dysfunction and Death in Parkinson’s Disease Remains Unknown

Irrespective of the etiopathogenesis of MRC deficiency in PD, given the neuronal dependency on mitochondrial respiration (Mary et al., 2012), it is likely to compromise the metabolic and bioenergetic status of neurons, and possibly other cell types, thereby contributing to cellular dysfunction and death in PD (Figure 2). The deleterious effect of MRC dysfunction was recently illustrated in a mouse model of CI deficiency specific to dopaminergic neurons, in which it was shown that isolated CI deficiency is sufficient to cause degeneration, beginning with axonal loss of function, with progressive levodopa-responsive parkinsonism (González-Rodríguez et al., 2021). While it is unknown whether similar effects take place in PD, CI deficiency is an established cause of neuronal dysfunction and death across multiple brain regions, including the dopaminergic SNc, in patients with mitochondrial disease (Tzoulis et al., 2016; Carelli et al., 2015; Flønes and Tzoulis, 2018). Therefore, it stands to reason that the MRC deficiency observed in PD is deleterious to neuronal function and survival and contributes to the neurodegenerative process (Figure 2). However, as with LP, there is not a high correlation between neuronal loss and reported MRC deficiency in PD (Dickson, 2012; Subrahmanian and LaVoie, 2021).

## Future Perspectives

Regardless of the variable and partly conflicting evidence, little doubt remains that MRC dysfunction, as a phenomenon, is associated with idiopathic PD. Specifically, CI deficiency in the SNc appears to be the most pronounced and consistently reported defect, whereas other complexes may also be affected, albeit to a lesser degree. In spite of having had this knowledge since 1989 (Schapira et al., 1989), the role of MRC dysfunction in PD, and whether it is deleterious, compensatory, or an innocent bystander, remain unknown. Several obstacles have prevented major breakthroughs in this field, including the inability to assess and track MRC function in the human brain during life, and the lack of reliable disease models. Below, we summarize three key knowledge gaps in this field, and propose potential strategies to explore and address them in future research.

### Is Mitochondrial Respiratory Chain Dysfunction a Pervasive Phenomenon in Parkinson’s Disease?

While referred to as a single entity, PD is a heterogeneous clinical syndrome, defined based on a constellation of phenotypical features (Bloem et al., 2021). Individuals with PD exhibit high phenotypic variability, on the basis of which several classification systems have been proposed, clustering subjects e.g., according to age of onset, combinations of motor and non-motor feature, rate of disease progression and variable treatment response (Espay et al., 2017; Greenland et al., 2019; Bloem et al., 2021). This phenotypical diversity has led to the hypothesis that biological subtypes of PD may exist, driven by different molecular mechanisms (Espay et al., 2017). Considering the clinicopathological heterogeneity of PD and striking variability in the reported MRC findings, it is not unreasonable to hypothesize that MRC dysfunction may characterize only a subgroup of individuals with PD. If true, this would imply that mitochondrial dysfunction underpins a distinct subtype of PD, and, at the same time, provide the opportunity to interrogate and decipher the role of mitochondrial dysfunction in the disease. With the appropriate markers, PD samples and cohorts could be stratified according to MRC deficiency, thereby increasing the signal-to-noise ratio in both basic and clinical research and increasing the chances for success, while moderating the need for large sample sizes. Exploring the pervasiveness of MRC dysfunction in PD should be feasible with today’s technology, but will require systematic characterization of large samples, preferably with accompanying clinical and environmental information.

### What Is the Etiopathogenesis of Mitochondrial Respiratory Chain Dysfunction in Parkinson’s Disease?

In terms of the etiology of MRC dysfunction, current evidence suggests that genetics may play a role, but this has not been adequately explored. While associations at the pathway level have been highlighted (Gaare et al., 2018; Billingsley et al., 2019), these studies were underpowered for detecting signal at the level of individual variants or genes. Moreover, they did not consider complex interactions between nuclear and mtDNA variation. Addressing these questions would require single-base resolution genetic data from larger populations, probably in the order of 5–10,000 samples (Gaare et al., 2018). Since such datasets are now available via international consortia, this should be feasible to assess. That said, genetics are unlikely to be the sole driver of MRC dysfunction in PD, and the potential role of environmental factor, including exposure to MRC-inhibitors, ought to be better resolved as multiple clinical cohorts of increasing sizes are being studied across most populations.

In terms of the mechanisms underlying the observed decrease in MRC complexes, evidence supports that this is, at least partly, mediated by somatic mtDNA changes. While we know that this type of mtDNA alterations can cause MRC deficiencies in models and other diseases, proving that this is the case in PD is no trivial challenge. Having no cell or animal models that recapitulate the pathogenesis of PD, answers ultimately rely on the study of postmortem brain tissue. Since MRC deficiencies occur in a mosaic distribution (Flones et al., 2017), this question must be addressed at a cell-specific level, e.g., by combining immunostaining with laser-microdissection. A handful of such studies have been reported in the SNc with results corroborating the link between mtDNA changes and MRC defects (Bender et al., 2006; Grünewald et al., 2016). Similar studies are warranted in other areas and cell-types showing MRC deficiencies in PD and assessing the full spectrum of mtDNA changes (deletions, copy number, point mutations) in each cell. A similar approach can be used to assess the transcriptomic profile of MRC-deficient and competent cells. If these deficiencies are mediated by mtDNA changes, it stands to reason that the expression of MRC subunits will be decreased in these cells.

### What Is the Downstream Impact of Mitochondrial Respiratory Chain Dysfunction in Parkinson’s Disease?

To understand the impact of MRC deficiencies in PD there is a need to combine experiments in appropriate models of MRC deficiency with cell-specific molecular signatures of the PD-brain. In this integrated approach, models can interrogate causality, while brain studies will establish relevance for the disease. Good models would reflect the type and magnitude of the MRC deficiencies observed without perturbing other biological systems. A paradigm of such a model was recently reported (González-Rodríguez et al., 2021). Cell-specific molecular studies of the PD-brain should differentiate between subtypes of neurons and/or glia, and distinguish between states of cell-specific pathology (i.e., presence, type and severity of MRC deficiency and α-synuclein pathology), so that specific molecular-pathological associations may be derived. Conducting such studies to full extent with current technology would be challenging. Immunostaining combined with laser-capture microdissection is a highly versatile, but also cumbersome approach. Moreover, the quality of derived omics, such as transcriptomics or proteomics, is often poor. Medium and high-throughput technologies (Nagy et al., 2020) are under rapid development and will hopefully provide us with the means to perform such studies at higher volume, efficiency, and precision.

To determine whether MRC dysfunction contributes to the initiation and progression of PD, there is a need to understand how it evolves over time, as well as if/how it is connected to disease progression. Doing so requires extra-neural and/or non-invasive brain markers of mitochondrial pathology. Currently, no imaging biomarkers have been shown to directly detect mitochondrial dysfunction, in the PD brain. A recently developed positron-emission tomography (PET) tracer for quantitative imaging of CI has shown promise in neurotoxic models of PD, but detected no changes in a human trial (Wilson et al., 2020). Even if the MRC itself cannot be directly assessed in the living brain, it may be possible to detect associated metabolic changes, measurable by e.g., phosphorus (31P) magnetic resonance spectroscopy (MRS), which assesses phosphorylated metabolites including NAD and ATP (Lu et al., 2014), and/or metabolomic analyses in cerebrospinal fluid. However, the potential of these powerful technologies has not been fully harnessed in PD. Furthermore, it is important to settle the question of whether signs of MRC deficiency can be detected in peripheral tissues of individuals with PD. The contradictory results of previous studies, in this regard, are consistent with a heterogeneous effect, where MRC dysfunction only occurs in a subpopulation of PD subjects. Addressing this heterogeneity will require studies in larger patient groups, employing multidisciplinary methodologies (e.g., immunodetection, functional assays, mtDNA assessment, and targeted metabolomics), and assessing interindividual variability and the potential for stratification of the examined cohorts.

In conclusion, two centuries since PD was first described, our pathophysiological insight still relies largely on phenomenology. Along with LP, MRC dysfunction is a phenomenon associated with PD, albeit less consistently, but whose potential role in disease initiation and progression remains poorly understood. We hope that future studies will provide the much-sought answers to these pertinent questions and begin to unwind the mystery of mitochondrial dysfunction in PD.

## Abbreviations

CI, complex I; CIV, complex IV; IHC, immunohistochemistry; LP, Lewy pathology; MRC, mitochondrial respiratory chain; MRS, magnetic resonance spectroscopy; mtDNA, mitochondrial DNA; PD, Parkinson’s disease; PET, positron-emission tomography; ROS, reactive oxygen species; SNc, substantia nigra pars compacta; TFAM, mitochondrial transcription factor A.

## Data Availability

The original contributions presented in the study are included in the article/Supplementary Material, further inquiries can be directed to the corresponding author.

## Abbreviations

CI, complex I; CIV, complex IV; IHC, immunohistochemistry; LP, Lewy pathology; MRC, mitochondrial respiratory chain; MRS, magnetic resonance spectroscopy; mtDNA, mitochondrial DNA; PD, Parkinson's disease; PET, positron-emission tomography; ROS, reactive oxygen species; SNc, substantia nigra pars compacta; TFAM, mitochondrial transcription factor A.
